# Complex Trophic Interactions in an Acidophilic Microbial Community

**DOI:** 10.3390/microorganisms10071340

**Published:** 2022-07-02

**Authors:** Guntram Weithoff, Elanor M. Bell

**Affiliations:** 1 Department Ecology and Ecosystem Modelling, University of Potsdam, 14469 Potsdam, Germany; 2 Berlin-Brandenburg Institute of Biodiversity Research, 14195 Berlin, Germany; 3 Australian Antarctic Division, Channel Highway, Kingston, TAS 7054, Australia; elanor.bell@awe.gov.au

**Keywords:** acid mine drainage, extremophiles, food web, heliozoa, intraguild predation, mining lakes, Rotifera

## Abstract

Extreme habitats often harbor specific communities that differ substantially from non-extreme habitats. In many cases, these communities are characterized by archaea, bacteria and protists, whereas the number of species of metazoa and higher plants is relatively low. In extremely acidic habitats, mostly prokaryotes and protists thrive, and only very few metazoa thrive, for example, rotifers. Since many studies have investigated the physiology and ecology of individual species, there is still a gap in research on direct, trophic interactions among extremophiles. To fill this gap, we experimentally studied the trophic interactions between a predatory protist (*Actinophrys sol*, Heliozoa) and its prey, the rotifers *Elosa woralli* and *Cephalodella* sp., the ciliate *Urosomoida* sp. and the mixotrophic protist *Chlamydomonas acidophila* (a green phytoflagellate, Chlorophyta). We found substantial predation pressure on all animal prey. High densities of *Chlamydomonas acidophila* reduced the predation impact on the rotifers by interfering with the feeding behaviour of *A. sol*. These trophic relations represent a natural case of intraguild predation, with *Chlamydomonas acidophila* being the common prey and the rotifers/ciliate and *A. sol* being the intraguild prey and predator, respectively. We further studied this intraguild predation along a resource gradient using *Cephalodella* sp. as the intraguild prey. The interactions among the three species led to an increase in relative rotifer abundance with increasing resource (*Chlamydomonas*) densities. By applying a series of laboratory experiments, we revealed the complexity of trophic interactions within a natural extremophilic community.

## 1. Introduction

Extreme habitats occur all over the planet from the deep sea to hot deserts, from cold dark caves to hydrothermal springs and many more. They differ in their type and degree of extremeness, but organisms require special adaptations to cope with each [1]. One type of extreme, aquatic habitat is extremely acidic lakes. Most natural water bodies, marine and freshwater, have a circum-neutral pH in the range of 6 to 8.5. Peat bogs are typically acidic (pH around 5–6), but extremely acidic water bodies have a pH < 2.8 (according to the classification of Nixdorf et al. [2]). Extremely acidic lakes can occur naturally, for example, due to volcanic activity [3], or be formed anthropogenically, for example, following the cessation of open-cast mining activities and acid mine drainage. A very low pH is often accompanied by high concentrations of (heavy) metal ions [4]. Under such harsh conditions, prokaryotes (bacteria and archaea) typically dominate the biota, and only a few eukaryotic species contribute to the biotic community [5,6,7,8,9]. Most of these eukaryotes are protists, for example, flagellated mixotrophs from the divisions chlorophyta, euglenophyta and heterokontophyta [10,11,12,13,14] or heterotrophs such as rhizopods, ciliates or heliozoa [15,16,17]. Acidophilic metazoans such a rotifers and crustaceans are rare, and only a few acidophilic species have been found [18,19,20].

Whereas the ecology and physiology of archaea, bacteria [4,21,22] and also some autotrophic/mixotrophic protists such as *Chlamydomonas* and *Ochromonas* have been intensively investigated [23,24], studies on their consumers, in particular their trophic interactions, are rare. To fill this gap, we studied the trophic interactions between the protistan top predator *Actinophrys sol* and its prey: the osmo-mixotrophic protist *Chlamydomonas acidophila* (a green phytoflagellate, Chlorophyta), the ciliate *Urosomoida* sp. (Hypotricha) and the metazoa *Cephalodella* sp. and *Elosa woralli* (Monogononta, Rotifera). Specifically, in a micro-scale experiment, we tested the short-term predation impact of *A. sol* on the ciliate and the rotifers depending on the nutritional status of the predator (starved, non-starved) and with and without accompanying algal food. In a population-dynamics experiment (meso-scale), we quantified the impact of *A. sol* on all three prey species. To further study the intraguild predation relationship between the common resource *Chlamydomonas* and the intraguild prey (*Cephalodella* sp.) and predator (*A. sol*), we ran a population-dynamics experiment at various levels of the common resource. Ultimately, we combined these results with those from previous studies [25] to produce two food web scenarios at different levels of productivity.

## 2. Materials and Methods

### 2.1. Organisms, Place of Origin and Culture Conditions

All organisms used in this study were isolated from two extremely acidic mining lakes (pH 2.6–2.8): *Actinophrys sol* [25], *Cephalodella* sp. (previously misidentified as *C. hodii*, [26]), *Elosa woralli* [27] and *Chlamydomonas acidophila* [28,29] from mining lake 111 and *Urosomoida* sp. [17] from Lake Goitsche, in the Lusatian region of Eastern Germany. Lake 111 is a small (0.11 km^2^ surface area) brown-coal-mining lake and is located in the East of Germany (Lusatia; 51°29′ N, 13°38′ E). The mean (maximum) depth is 4.6 (10) m, and the lake is thermally stratified during summer [10]. All organisms were reared in a culture medium simulating the extreme pH (2.65) and the high mineral concentrations of Lake 111 [30]. This medium was particularly rich in iron (2.6 mM) and sulphate (19.74 mM). The nutrient concentrations were 0.44 mM nitrogen as potassium nitrate and ammonium sulphate and 6.5 µM phosphorus as potassium di-hydrogen phosphate. Heliozoan stock cultures were maintained with a mixed diet of *Chlamydomonas acidophila* and the two rotifer species as food sources. The rotifers and the ciliates were also cultured with *Chlamydomonas* as food. The mean individual cell volume of *A. sol* was 34,000 µm^3^ (±17,600 µm^3^, standard deviation) and was strongly dependent on feeding history. *Urosomoida* sp. has quite variable cell volume. The mean cell volume in our experiments was 2900 µm^3^ (±1900 µm^3^, standard deviation) and was lower than in a previous study 3800 µm^3^ (±1700 µm^3^, standard deviation) [16]. The two rotifer species ranged between 50,000 and 100,000 µm^3^, also dependent on their feeding history [26].

### 2.2. Experimental Set-Up

We used three experiments to investigate the trophic impact of *Actinophrys* on its prey. All of them were run in a climate chamber at 20 °C and with a light–dark cycle of 16:8 h.

(1) In a micro-scale experiment, we studied the predation using microtitre plates with a volume of 200 µL. Heliozoa were isolated from healthy stock cultures. Four treatments were applied: (i) a control, i.e., *A. sol* without any further treatment, (ii) *A. sol* plus 100,000 cells mL^−1^ *Chlamydomonas* (~2.1 µg C mL^−1^), (iii) *A. sol*, which were starved for 48 h prior to the experiment, and (iv) starved *A. sol* as in (iii) plus 100,000 cells mL^−1^ *Chlamydomonas* (~2.1 µg C mL^−1^). For each treatment, 24 to 36 *A. sol* were transferred separately into the wells of a microtitre plate, either filled with sterile medium or with the *Chlamydomonas* suspension. Subsequently, two individual prey items were added from a single prey species (either *Cephalodella* sp., *E. woralli* or *Urosomoida* sp.). After 24 and 48 h, the microtitre plates were examined under a binocular microscope, and the survival of the rotifers and *Urosomoida* sp. was recorded. Since some rotifers either produced an egg or died after 24 h, and since *Urosomoida* sp. grew during the experimental period, results are shown only for the first 24 h time interval. Additionally, in the *Urosomoida* sp. experiments, 12 control treatments were set up with two ciliates and no Heliozoa to quantify the growth without predation. For the analysis, the difference between the number of ciliates in treatments containing *A. sol* and the mean of the treatments without *A. sol* was calculated. For all three predator–prey pairs, three independent sets were run.

(2) A second, meso-scale experiment was run to investigate the impact of *A. sol* on their prey using natural *A. sol* densities [16,24]. One problem encountered during the experiments was our ability to provide sufficient *Chlamydomonas* food to promote rotifer growth without negatively influencing *A. sol*, since the growth rate of *A. sol* is known to decrease when food particle densities are too high [25]. To address this, 500 Heliozoa plus 500 rotifers were added to a 100 mL *Chlamydomonas* suspension with a density of 8000 cells mL^−1^ (~0.22 µg C mL^−1^). This density was found to be suitable for the growth of *A. sol* and supported the growth of *E. woralli*. However, it was below the resource density threshold for *Cephalodella* sp. [26,31]. Three parallel flasks were set up with and without Heliozoa to compare the response of the prey to their predator. In the analogous meso-scale experiment with *Urosomoida* sp. (starting with 5000 animals per 100 mL), glucose (20 mg C L^−1^) was added to the medium to support bacterial growth to provide food for the ciliates. Previous experiments showed that single-celled bacteria do not inhibit or promote the growth of *A. sol* [25]. This experiment was run in conical flasks that were gently shaken. Every two days, 12 mL were removed from each flask, fixed with Lugol’s iodine and acidified with sulphuric acid to avoid iron precipitation. The volume removed was replaced with fresh medium. The sub-samples were subsequently examined by inverted light microscopy (Thalheim, Pulsnitz, Germany).

(3) Since we did not control for the resource level in the second experiment, a third experiment was run in which we used five different resource levels and kept them constant. As the intermediate species, we chose *Cephalodella* sp., because it was affected more by the predation of *A. sol* than *E. woralli*. In six-well microtitre plates, we filled three parallel wells with food suspensions of 5000, 10,000, 20,000, 40,000 and 100,000 cells mL^−1^ *Chlamydomonas* and added 10 individuals of *A. sol* and 10 of *Cephalodella* sp. Each day, most of the food suspension was carefully removed with a fine glass pipette, the heliozoa and rotifers were counted in the remaining medium, and then fresh food suspension of the target concentration was resupplied. After eight days, the experiment was terminated by adding Lugol’s iodine, and the final densities of the heliozoa and rotifers were determined. 

### 2.3. Statistical Analysis

For the statistical analysis of the micro-scale experiments with rotifers, the proportion of *A. sol* having eaten a rotifer in relation to the total number of *A. sol* was calculated for each of the three runs separately. These proportions were arcsin-transformed and then analysed with an ANOVA, with treatment and species as independent factors. For the experiments with the ciliate, a different procedure had to be applied. The ciliates grew during 24 h, so we compared the number of ciliates without *A. sol* with the ciliate numbers with the predator. Therefore, we calculated the mean number of ciliates from the 12 predator-free controls and subtracted it from the mean number of ciliates with the predator. Mean differences among treatments were compared using a univariate ANOVA. To compare prey abundances with and without Heliozoa in the meso-scale experiments, a general linear model (ANOVA) with repeated measures was applied. All analyses were performed using SPSS, version 26 (IBM, New York, NY, USA).

## 3. Results

### 3.1. Micro-Scale Experiment

#### 3.1.1. Rotifers

**Figure 1:**
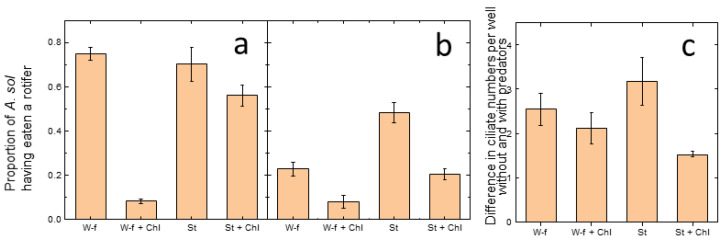
Feeding of *Actinophrys sol* on rotifers and ciliates after 24 h. Proportion of *A. sol* having eaten (**a**) a *Cephalodella* sp. individual or (**b**) an *Elosa woralli* individual. (**c**) Difference in ciliate numbers without and with *A. sol* per well. W-f, well-fed; St, staved; + Chl, 100,000 cells mL^−1^, *Chlamydomonas* added. Mean ± standard error, *n* = 3 sets of 24–30 individual wells. For rotifers, both the treatment and the species effect were significant, *p* < 0.001, as was the species x treatment interaction (*p* < 0.001). For ciliates, no treatment effect was found (*p* = 0.07).

*Actinophrys sol* preyed on both rotifer species in all four treatments; however, predation varied among treatments and species (Figure 1): The proportion of heliozoa having eaten an individual *Cephalodella* sp. was higher than those having eaten an *Elosa woralli* individual (F = 89.4, df = 1, *p* < 0.001), suggesting a higher predation pressure on *Cephalodella* sp. For both rotifer species, a clear treatment effect was found (F = 57.5, df = 3, *p* < 0.001), and the proportion of heliozoa having eaten a rotifer was lowest in the treatment with non-starved *A. sol* plus *Chlamydomonas* (Tukey post-hoc test *p* < 0.001). The effect of the additional food source *Chlamydomonas* was overruled by pre-starvation of the animals, because predation on rotifers was high in the presence of *Chlamydomonas* when *A. sol* was starved prior to the experiment. Despite the similarities in the effect on the rotifers, the overall pattern differed among the two species (interaction of treatment * species, F = 11.6, df = 3, *p* < 0.001), for example, in the response to starvation.

#### 3.1.2. Ciliates

Overall, the variation within treatments in the response of the ciliates to heliozoan predation was high, and no significant differences among treatments were found (F = 3.5, df = 3, *p* = 0.07). On average, over all treatments, two more ciliates were found in the predator-free environment, suggesting a strong predation pressure on the ciliates (Figure 1c and Supplementary Materials, Table S1).

### 3.2. Meso-Scale Experiment

#### 3.2.1. Rotifers

**Figure 2:**
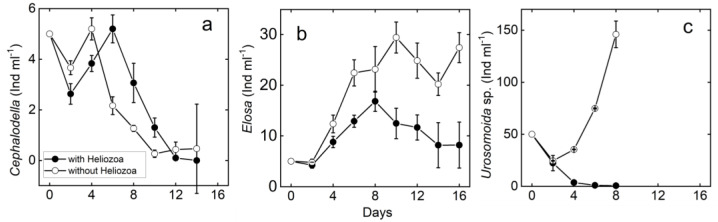
Time course of Heliozoan prey (**a**, *Cephalodella* sp.; **b** *Elosa woralli*; **c**, *Urosomoida* sp.) with and without *A. sol*. Mean ± standard error (*n* = 3). Abundance of both rotiferan prey differed between treatments (*p* = 0.03 for both) and also for ciliates (*p* << 0.001). Note different y-axis scales.

The results from the meso-scale experiment were strongly species-specific (Figure 2). Although the abundance of *Cephalodella* sp. was significantly different between treatments with and without *A. sol* (F = 11.37, df = 1, *p* = 0.03), their abundance in the treatment with *A. sol* over the experimental period was sometimes higher and sometimes lower than in the treatment without *A. sol*, showing no clear predation effect. In neither of the *Cephalodella* sp. treatments did the rotifers grow positively after day six, likely due to low *Chlamydomonas* densities. In contrast, *E. woralli* grew in both treatments or remained relatively constant when taking the experimental dilution into account. *E. woralli* abundances were lower when *A. sol* was present (F = 10.05, df = 1, *p* = 0.03), indicating a significant predation pressure on *Elosa* (Figure 2b). The growth of *A. sol* was on average lower in the treatment with *E. woralli* (Supplementary Materials, Table S2) than in the *Cephalodella* sp. Thus, *E. woralli* seems to be the less favourable food source.

#### 3.2.2. Ciliates

The most pronounced predation pressure observed was by *Actinophrys sol* on *Urosomoida* sp. (F = 284.09, df = 1, *p* < 0.001). Within 6–8 days, *A. sol* drove the *Urosomoida* sp. population almost to extinction (Figure 2). In the absence of *A. sol*, *Urosomoida* sp. grew at a rate of approximately 0.36 d^−1^. Thus, the results from the ciliate micro-scale experiments were reinforced with the meso-scale experiments (Figure 1c and Figure 2c).

### 3.3. Resource Level Experiment

**Figure 3:**
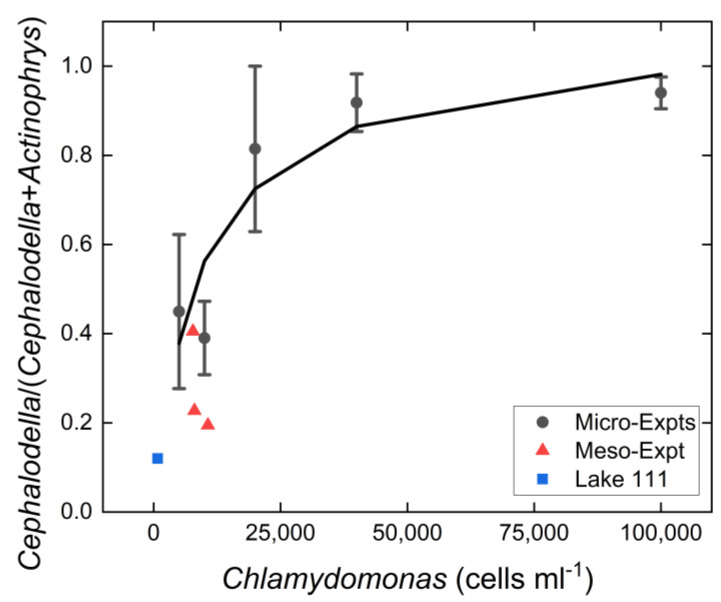
Relative contribution of *Cephalodella* sp. to total consumer abundance at varying productivity levels. Data were combined from the micro-scale experiments (Micro-Expts) mean ± standard error, the meso-scale experiments (Meso-Expts) and field data for mean abundances over a three-year observation period (field data from [25]). Solid line, trend curve fit.

Increasing productivity applied as increasing numbers of *Chlamydomonas* significantly enhanced the growth of consumers. Mean consumer (*A. sol* + *Cephalodella* sp.) abundance increased from 3.1 mL^−1^ in the 5000 cells mL^−1^ treatment to 402 mL^−1^ in the 100,000 cells mL^−1^ treatment. This increase was almost exclusively due to the increase in *Cephalodella* with increasing productivity (from 1.5 mL^−1^ at 5000 cells mL^−1^ to 382 mL^−1^ at 100,000 cells L^−1^), whereas the abundance of the heliozoa exhibited a moderate peak at 10,000 cells mL^−1^ and ranged between 1.4 and 2.9 mL^−1^ over the whole productivity gradient (Supplementary Materials, Table S3). These different numerical responses led to a pronounced increase in the relative contribution of *Cephalodella* sp. to total consumer numbers (Figure 3). 

**Figure 4:**
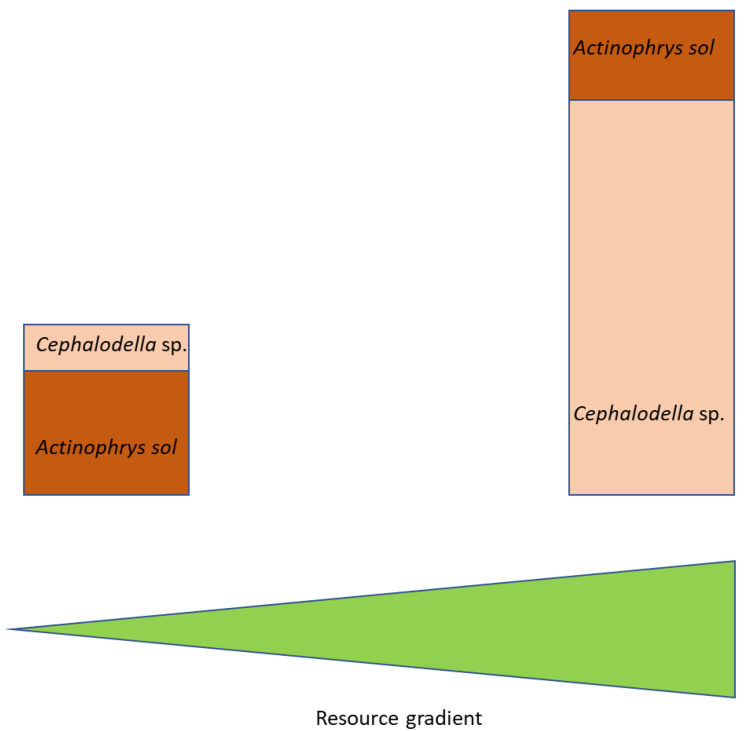
Schematic representation of the relationship of resource availability and consumer and predator abundance.

Combining these findings with those from the meso-scale experiment, as well as mean seasonal abundance data for *Cephalodella* sp. and *A. sol* in their lake of origin, the extremely acidic Lake 111, [25], a consistent pattern occurs: a higher contribution of *A. sol* to total consumer numbers at low resource concentrations and a higher contribution of *Cephalodella* sp. at high resource concentrations. Summarizing these results, two scenarios are apparent: a low-productivity scenario with a dominant intraguild predator and a high-productivity scenario with a dominant intraguild prey species (Figure 4). 

## 4. Discussion

We observed clear predation pressure on all three natural prey species tested, emphasising the status of *A. sol* as top predator in the planktonic food web in an extremely acidic lake, Lake 111 [25]. Whereas the ciliates were substantially smaller than the Heliozoa (~10% of *Actinophrys sol*), the rotifers were larger in volume than their predator. *A. sol* manage to ingest prey larger than themselves by their particular feeding mode: when large prey particles attach to the axiopods, they are immobilised [32,33], transported to the cell body, enveloped and digested [34,35,36]. Despite similarities in prey capture mechanism for each of the three prey species, the predator–prey interactions are complex and lead to context-dependent community compositions.

### 4.1. Predation on Rotifers

Both rotifer species were eaten by *A. sol*, but the predation pressure was higher on *Cephalodella* sp. than on *E. woralli*. Since heliozoa are non-motile predators, the encounter probability mainly depends on the movement behaviour of their rotiferan prey. From live microscopical observations, it is difficult to pinpoint the differences in movement behaviour between the two rotifer species, but one may speculate that *E. woralli* lowered its capture probability because of a slightly different swimming behaviour. In the micro-scale experiment, the density of heliozoa (1 individual in 200 µL, which equals to 5000 ind L^−1^) was comparable to the maximum densities observed in Lake 111 in summer of 4000 to 7000 ind L^−1^ [25]. However, the densities of both rotifer species used in our experiment were much higher than those observed to occur naturally in Lake 111 [27]. This may have led to an overestimation of the absolute predation pressure. Nevertheless, the relative relationships among prey species and treatment are likely consistent. Estimations of natural predation pressure in the field are difficult to make. The abundance maxima in Lake 111 differ between the two rotifers; *Elosa woralli* is dominant in the upper part of the water column and *Cepahlodella* sp. in the lower part. Thus, the maxima of *E. woralli* (lower predation risk) and *A. sol* overlap in the field [25,27]. However, the vertical distribution of the three species cannot be explained by a single factor, because resource quantity and quality and temperature all play a role [37,38,39].

The strength of the predation pressure on the rotifers decreased when *Chlamydomonas* was supplied at a density of 100,000 cells mL^−1^. This additional food reduced the predation on rotifers not only by “diluting” them, but also by clogging the heliozoa’s axiopods and reducing their total food uptake. This mechanism has been described by Bell et al. and can lead to reduced growth of *A. sol* [25].

The interplay between resource supply and predation was further supported by the meso-scale experiment. For *E. woralli*, the resource level was obviously above the threshold for growth, since in both treatments, *E. woralli* increased in number at the start of the experiment. With predators, *E. woralli* declined after a few days, and their abundance was controlled at a lower level. For *Cephalodella*, the outcome was different: without heliozoa, the abundance of *Cephalodella* was higher than with their predator during the initial days of the experiment, which led to a faster depletion of their algal food source and the decline of *Cephalodella*. With heliozoa, the overall pattern was similar, but initially lower *Cephalodella* abundances likely led to a slower decline of their algal food source and consequently of *Cephalodella* themselves. To further elucidate the relation between heliozoa, *Cephalodella* and their common food resource, *Chlamydomonas acidophila*, we performed the third, intraguild predation experiment (see Section 3.3).

### 4.2. Predation on Ciliates

*Urosomoida* sp. is a fast-growing ciliate that grew during the experiment and compensated partly for its predation losses. Consequently, in the micro-scale experiment, the difference in ciliate numbers with and without the heliozoan predator was quantified. Although the overall pattern resembled that of the rotifer experiment, differences among treatments were not significant (*p* = 0.07). Nevertheless, in both ciliate experiments, a clear and substantial predation pressure was found, demonstrated by the differences in ciliate abundances with and without predators. Thus, the ability of ciliates and rotifers to thrive under extremely acidic conditions does not release them from predation pressure. Acidophilic or acidotolerant ciliates have been found in other extreme habitats, but they are typically not the dominant heterotrophs and play a minor role in the food web [9,10,17,40].

### 4.3. Food Web Implications

The trophic relationship between the heliozoa, the ciliates/rotifers and their common prey, the phytoflagellate *Chlamydomonas acidophila*, represent a special case of intraguild predation. The term intraguild predation refers to a three-way trophic relationship between a shared resource species (in this case *Chlamydomonas acidophila*); a consumer of this resource, the intraguild prey (the ciliate and/or the rotifers, in particular *Cephalodella* sp.); and an omnivorous consumer, the intraguild predator (*Actinophrys sol*) feeding on both the shared resource and the intraguild prey. Results from mathematical modelling and laboratory experiments have demonstrated that enrichment of resources facilitates the intraguild predator and reduces the intraguild prey or even drives the intraguild prey to extinction [41,42]. These interactions result from the specific traits of the species, when the intraguild prey is the superior competitor over the intraguild predator at low productivity, but enrichment of their common resource allows the intraguild predator to feed upon both the resource and the prey and thereby exert a higher predation pressure on the intraguild prey [41]. In the present case, these relationships are different. A high abundance of the shared prey, *C. acidophila*, reduces the growth rate of the intraguild predator (*A. sol*) by clogging their axiopods and increases the growth rate of the intraguild prey (the ciliate and/or rotifers), leading to the dominance of *Cephalodella* sp. Based on these results, we can predict two scenarios, one for low- and one for high-productivity situations (Figure 4):

(1) At low productivity, the intraguild predator can suppress the intraguild prey and simultaneously make use of the shared resource.
(2) At high productivity, the growth of the intraguild predator is reduced due to clogging of the axiopods by too many small prey items. This overload releases the intraguild prey (here: *Cephalodella* sp.) from predation pressure, allowing it to grow to high abundances.

This example of naturally occurring intraguild predation can only be explained when taking into account the specific nature of the interacting species.

### 4.4. Comparison with Circum-Neutral Habitats

In circum-neutral habitats (pH 6–8.5) such as lakes and oceans, the relative roles of ciliates, rotifers and heliozoa differ from those in acidic lakes. Ciliates contribute little to the food web in extremely acidic lakes [9], but they are important players in the microbial loop in lakes [43,44,45] and oceans [46,47]. There, they are consumed mainly by crustaceans (copepods and cladocerans) and to a lesser extent by rotifers. Rotifers in turn are preyed upon by a wide variety of invertebrates ranging from predatory rotifers (*Asplanchna*) and crustaceans to insects but also by fish larvae. The mean abundance of *A. sol* in Lake 111 is in the range of the abundance of heliozoa in lakes and coastal oceans [48,49], but episodic higher abundances in mining lakes have been observed [50]. Since the other groups of micro-zooplankton increase in abundance in circum-neutral lakes, the relative importance of heliozoa in these food webs decreases [51]. Thus, the trophic interactions described here differ from what is known from circum-neutral habitats and are of specific relevance for understanding the food web structure in such extreme habitats.

## Data Availability

Data are provided as Supplementary Materials.

## Supplementary Materials

Supporting information can be downloaded at: https://www.mdpi.com/article/10.3390/microorganisms10071340/s1, Supplementary_Data__Weithoff&Bell.xlsx (containing Table S1, microscale experiment; Table S2, mesoscale experiment and Table S3, resource level experiment).
